# Issues occupying our minds: Nomenclature of autoimmune blistering diseases requires updating, pemphigus vulgaris propensity to affect areas adjacent to natural body orifices unifies seemingly diverse clinical features of this disease

**DOI:** 10.3389/fimmu.2022.1103375

**Published:** 2022-12-19

**Authors:** Marian Dmochowski, Magdalena Jałowska, Monika Bowszyc-Dmochowska

**Affiliations:** ^1^ Autoimmune Blistering Dermatoses Section, Department of Dermatology, Poznan University of Medical Sciences, Poznan, Poland; ^2^ Section of Cutaneous Histopathology and Immunopathology, Department of Dermatology, Poznan University of Medical Sciences, Poznan, Poland

**Keywords:** autoimmune bullous diseases, nomenclature, pemphigus vulgaris, natural body, orifices

## Abstract

In this conceptual analysis, we present our concepts on two issues regarding autoimmune bullous diseases (AIBD), namely (i) current nomenclature of AIBD requires updating by incorporating molecular data and (ii) pemphigus vulgaris (PV) “likes” areas adjacent to natural body orifices. The problem of inadequacy of the currently used nomenclature was noticed recently by Zillikens, who proposed to form a group with the task of updating it. The early efforts by Dmochowski to update this nomenclature happened to be a daunting task. Nevertheless, the ideal nomenclature should retain the bulk of clinical data, which generations of dermatologists are accustomed to, including triggers if known, and incorporate molecular data revealing targets of autoimmune response and immunoglobulin isotypes involved. The natural body orifices affected by PV were previously described in numerous publications. However, these openings are described separately in these publications. Here, Dmochowski comes up with an intellectual concept that this propensity of PV unifies seemingly diverse clinical features of this disease.

## Introduction

We participated in two meetings organized by the people from the Department of Dermatology, University of Lübeck led by Prof. Detlef Zillikens, namely International Pre IID 2013 Meeting on Autoimmune Bullous Diseases (MD) and Scientific Conference of International Pemphigus & Pemphigoid Foundation in 2017 (MD, MBD). We were impressed by the quality of experimental research conducted in this department and the extraordinary hospitality we enjoyed there. Saddened deeply by the news that Prof. Detlef Zillikens, one of the giants of modern dermatology, passed away prematurely, we decided to present two issues we are preoccupied with for years. The ideal nomenclature of autoimmune bullous diseases should retain the bulk of clinical data, which generations of dermatologists are accustomed to, including triggers if known, and incorporate molecular data revealing targets of autoimmune response. The intellectual concept conceived by Dmochowski which he now promotes is that propensity of pemphigus vulgaris (PV) to affect natural body orifices unifies seemingly diverse clinical features of this disease.

## Current nomenclature of autoimmune blistering dermatoses requires updating by incorporating molecular data

**Figure 1:**
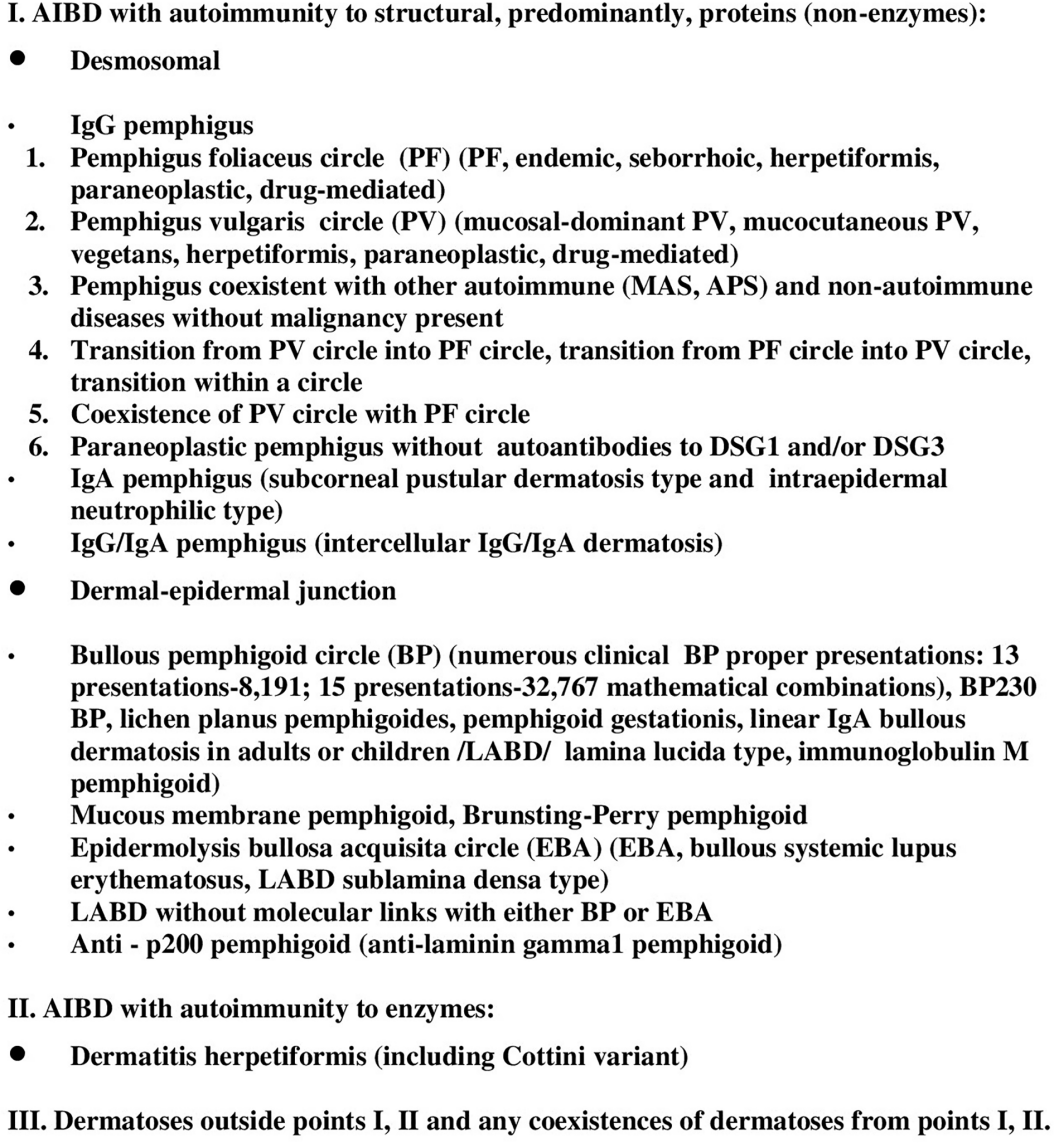
Current version of nomenclature of AIBD based on clinical-molecular features according to M. Dmochowski.

It was Prof. Zillikens initiative communicated on September 30, 2021 to organize a working group within the activities of EADV task force on AIBD on the novel nomenclature of AIBD. He thought that the updated nomenclature should incorporate molecular data on the pathogenesis of those disorders. Dmochowski eagerly responded as he proposed his nomenclature of AIBD along this idea as early as 2003 (1). Unfortunately, this was published in the Polish language journal that was at that time unindexed by MEDLINE and thus went unnoticed by the wider dermatology community. Its newest version showing novel data (2) is shown in **Figure 1**. Hopefully, it can serve as a contribution to considering the issue further. In total, just nine people responded from Europe, Israel and Singapore. The only online meeting took place on January 25, 2022 when group members were able to voice their opinions on the draft of new nomenclature put forward by Prof. Zillikens. For example, the nomenclature like pemphigus (IgG anti-DSG3) and further along that line was proposed in the draft. Still, in our opinion, there were important caveats in that draft.

**Figure 2:**
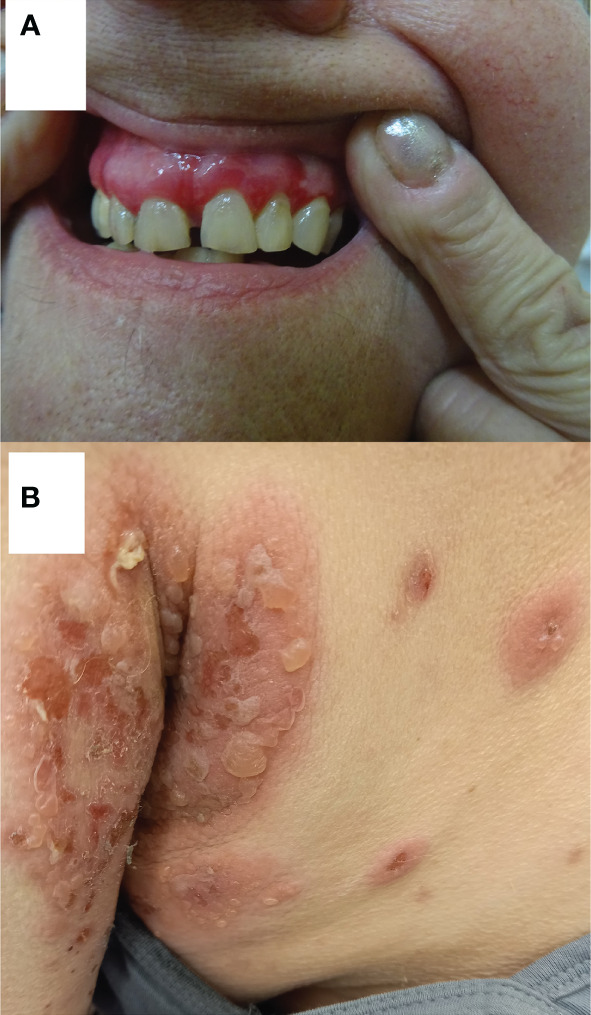
A middle-aged female with oral and cutaneous lesions plausibly related to nivolumab therapy for melanoma and mRNA vaccination for COVID-19 who could be diagnosed as mucous membrane pemphigoid/bullous pemphigoid (IgG anti-BP180 NC16A). Stubborn desquamative gingivitis **(A)**. Itchy urticarial and blistering lesions on armpit skin **(B)**. Reprinted/adapted with permission, Dmochowski M. Autoimmune bullous diseases associated with malignancy. 31st EADV Congress: Designing the Future of Dermatology and Venereology. Italy: Milan (2022) (3).

Recently we followed-up a female middle-aged patient (**Figure 2**) with a mucocutaneous disease showing stubborn desquamative gingivitis and widespread itchy urticarial and bullous cutaneous lesions that plausibly were triggered by the treatment with nivolumab, an immune checkpoint inhibitor, for melanoma (3). The oral lesions appeared first on nivolumab treatment, whereas cutaneous lesions followed soon after the third dose of mRNA vaccine for COVID-19. The multiplex ELISA revealed grossly elevated level of anti-BP180 NC16A antibodies whereas IgG antibodies to laminin 332 were not detected with mosaic indirect immunofluorescence. The problem of nomenclature in this case is that the stubborn desquamative gingivitis would suggest the diagnosis of mucous membrane pemphigoid (MMP), whereas widespread cutaneous lesions would suggest bullous pemphigoid (BP). The rational diagnosis in this case would be, along the ideas put forward by Prof. Zillikens, mucous membrane pemphigoid/bullous pemphigoid (IgG anti-BP180 NC16A) abbreviated conveniently to MMP/BP (IgG anti-BP180 NC16A). Obviously, such cases evade the nomenclature of the nosological system currently used. Therefore, our opinion is that the ideal nomenclature should retain the clinical data, including triggers if known, combined with molecular data revealing targets of autoimmune response (4) and immunoglobulin isotypes involved. The nosology of AIBD should be both succinct and precise, and abbreviations/acronyms should be both catchy and user-friendly. These postulates seem to be trivial, but they should not be ignored. It should be noted in this respect that certain disease names coined “ages ago”, like epidermolysis bullosa acquisita (EBA) or dermatitis herpetiformis (DH), are controversial now (5). The term EBA might wrongly suggest that epidermal lysis is taking place in this disease, whereas the term DH might imply a role of infection with herpes viruses in the pathogenesis of DH, which has never been proven to exist. Perhaps then, the term “gluten-sensitive IgA/neutrophil-mediated dermatitis” (the acronym GAND would sound nicely) suggested by us for the dermatosis described by Louis Adolphus Duhring would be more understandable for non-dermatologic medical/pharmaceutical/biotechnological community and lay public alike (6). The more recent term “paraneoplastic pemphigus” is also misleading as malignancy can coexist, albeit infrequently, with any AIBD with autoimmunity to predominantly structural proteins, regardless it has the word “paraneoplastic” in its name or not (7). The term “pemphigoid gestationis” which replaced the archaic term “herpes gestationis” unfortunately is also a misnomer, because this condition can manifest itself not only in pregnancy, but also in the postpartum period.

In the future, such a nomenclature incorporating molecular data should be particularly useful in the process of selecting AIBD patients for personalized therapies aiming at correcting exclusively patient-specific autoimmune responses.

## Pemphigus vulgaris “likes” areas adjacent to natural body orifices: An intellectual concept unifying its seemingly diverse clinical features

Decades ago, at times when immunological methods of diagnosing pemphigus diseases were not as readily available as there are nowadays, the patients might have been referred to university dermatologists while on later stages of pemphigus, namely full-blown disease. Patients might have had spectacular lesions covering large parts of the body at the time of the referral, thus lacking initial presentations around natural body orifices. Therefore the propensity of PV lesions at early stages of the disease to natural body orifices might have gone unnoticed by dermatologists of that time. The initial observations on that topic presenting monocentric clinical-laboratory experience were published by Dmochowski in 2007 in a Polish language journal again not covered by MEDLINE (8). Since then a number of papers and conference presentations containing relevant clinical-laboratory iconography that expand on that idea were prepared by Dmochowski et al. (9–15).

**Figure 3:**
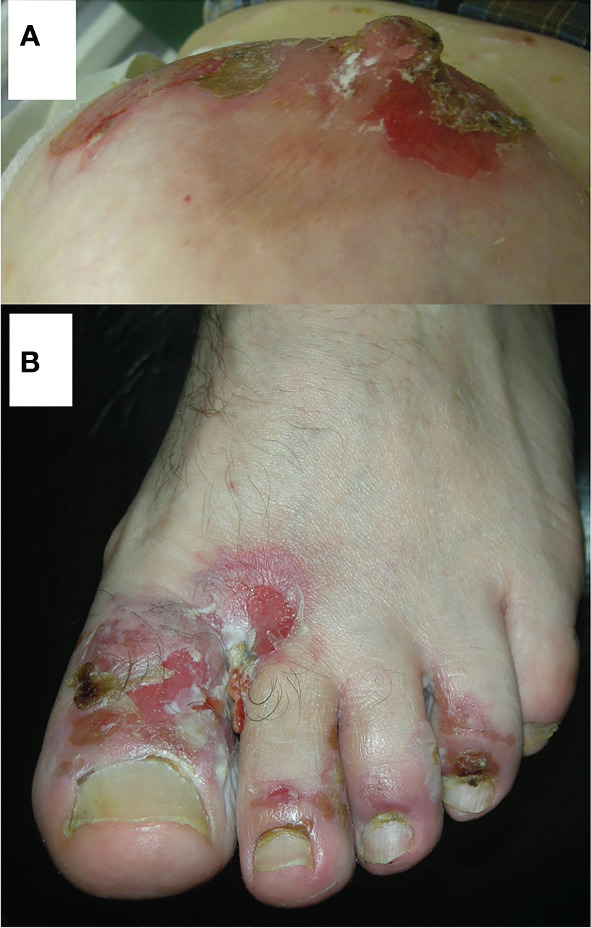
Representative PV patients having pemphigus lesions in less obvious locations adjacent to natural body orifices. A middle-aged female with cutaneous variety of PV having pemphigus lesions around the nipple of the breast **(A)**. Reprinted/adapted with permission, Dmochowski M. Pęcherzyca zwykła „lubi” okolice naturalnych otworów ciała/Pemphigus vulgaris “likes” areas around natural body orifices. X konferencja naukowo-szkoleniowa stowarzyszenia dermatologów wojska polskiego skóra 2022. Poland: Gdańsk (2022) (14). A middle-aged male with mucocutaneous variety of PV having pemphigus lesions on proximal nail folds of the toenails **(B)**. Reprinted/adapted with permission, Bowszyc-Dmochowska M, Pietkiewicz P, Gornowicz-Porowska J, Bartkiewicz P, Dmochowski M. Involvement of nail apparatus in pemphigus vulgaris in ethnic Slavs. 1st Congress of Trichoscopy. Symposium “All about hair & nails”. Poland: Warsaw (2018) (16).

We think now that the areas around the natural body orifices (**Figure 3**) should be considered predilection sites/sites of privilege for PV. Moreover, most importantly, Dmochowski realized that the tendency of PV lesions to affect those sites should be considered a feature unifying diverse clinical presentations of PV. These sites are the scalp, the area around the medial canthus, the concha of the auricle and the external auditory canal, the anterior nostrils, lips, the nipple of both female and male breast, the umbilicus, sacral/pilonidal/spinal dimples, anus, and external female and male genitalia, as well as the periungual areas of fingernails and toenails in any combinations. PV lesions can even affect the palmar surface of hands, where the openings of acrosyringia of eccrine sweat gland are abundant, manifesting themselves as cheiropompholyx (17). The patient with severe mucocutaneous PV presenting also cheiropompholyx followed-up by us had no H+E examination of palmar lesions done that would reveal acantholysis there. Nevertheless, those lesions cleared up during immunosuppressive therapy suitable for treating PV and never appeared again. There are data that DSG1 is expressed in the eccrine glands, therefore it may be an explanation for this clinical feature (18). Obviously, the issue of cheiropompholyx in relation to the severity of PV requires further proof and patients’ cohorts, ideally coming from diverse ethnicities, should be studied before arriving at the conclusion. Our opinion is that periorificial lesions should be a clinical hint for the clinicians of various specialties that they are dealing with a disease that requires imaging and biochemical-molecular immunopathological diagnostics and further patient management appropriate for the diagnosis of PV. The key question why PV “likes” the vicinity of natural body orifices requires an experimental explanation. The speculative explanations might be ideas that mechanosensing phenomena altering the expression and function of the desmosomal components (19) preferentially take place in such areas exposed to the intensive friction or that non-canonical functions of antibodies (20) more robustly manifest themselves in those sites. Mechanosensing describes the ability of a cell to sense mechanical cues of its microenvironment, including not only all components of force, stress, and strain but also substrate rigidity, topology, and adhesiveness (21). Therefore, mechanosensing may be relevant in the context the loss of adhesion characterizing PV, particularly in conjunction with the data that desmosomes are not merely structural proteins but also mediate keratinocyte transmembrane signalling (22). Non-canonical functions of antibodies such as antibody-mediated catalysis, receptor agonist activity of antibodies, antibodies acting as carriers and chaperones, and above all cell signalling modulated by antibodies (23) might be particularly relevant in PV at natural body orifices in relation to a periorificial microbiome. To sum up, we suspect that the microenvironment at any periorificial site facilitates the development of PV lesions.

## Author contributions

MD contributed to the conception and design of the article. MD wrote the first draft of the manuscript. MJ, MD, MB-D contributed to the writing—review, and editing. The formal analysis was done by MJ, MB-D. All authors contributed to the article and approved the submitted version.
